# Effect of iron content on the tolerability of prenatal multivitamins in pregnancy

**DOI:** 10.1186/1471-2393-8-17

**Published:** 2008-05-15

**Authors:** Patricia Nguyen, Alejandro Nava-Ocampo, Amalia Levy, Deborah L O'Connor, Tom R Einarson, Anna Taddio, Gideon Koren

**Affiliations:** 1 Department of Pharmaceutical Sciences, Leslie Dan Faculty of Pharmacy, University of Toronto, Toronto, Canada; 2 Motherisk Program, Hospital for Sick Children, Toronto, Canada; 3 Department of Epidemiology, Ben Gurion University, Be'er Sheva, Israel; 4 Department of Nutritional Sciences, Faculty of Medicine, University of Toronto, Toronto, Canada

## Abstract

**Background:**

Gastrointestinal irritability can deter pregnant women from starting or continuing prenatal multivitamin supplementation. In a previous study, suboptimal tolerability was observed among pregnant women taking a large tablet (18 mm × 8 mm × 8 mm) multivitamin with high elemental iron content (60 mg as ferrous fumarate). The objective of the present study was to compare rates of adherence and reported adverse events among pregnant women who were randomized to commence supplementation with a small-tablet prenatal multivitamin, containing either low or high iron content.

**Methods:**

Pregnant women who called the Motherisk Program (Hospital for Sick Children, Toronto) and had not started taking or had discontinued any multivitamin due to adverse events were included in this prospective, randomized, open-label, 2-arm study. Women were randomized to take a small-size (16 mm × 9 mm × 4 mm), low elemental iron content (35 mg as ferrous fumarate) multivitamin ('35 mg' group); or a small-size (5 mm radius, 5 mm thickness), high elemental iron content (60 mg as ferrous sulphate) multivitamin ('60 mg' group). Follow-up interviews documented pill intake and adverse events. Rates of adherence and adverse events were compared between groups using chi-squared tests and Kaplan-Meier survival curves.

**Results:**

Of 167 randomized women, 92 in the '35 mg' group and 75 in the '60 mg' group were included in the analysis. Despite ideal conditions and regular follow-ups, mean adherence based on pill intake recall, in both groups was approximately 50%. No statistically significant difference was detected in proportions of women who actually started taking either multivitamin. Among those who started, no difference was detected in rates of adherence or reported adverse events.

**Conclusion:**

The present results suggest that iron content is not a major determinant of adherence to prenatal multivitamins. Combined with our previous study, tablet size may be the more definitive factor affecting adherence.

## Background

Multivitamin-mineral supplementation is recommended before and during pregnancy to ensure adequate intake of several key nutrients including folate, iron, and more recently vitamin D – all of which have importance among pregnant women, women of childbearing age, and during fetal development [1-6]. Folic acid supplementation has been well documented to reduce the risk of neural tube defects (i.e. spina bifida) [7-9], and emerging evidence suggests that folic acid-containing multivitamin supplements are associated with reducing the risk of other malformations and certain pediatric cancers [10-13]. Furthermore, as pregnancy progresses, body size increases, alongside the nutritional requirements of the developing fetus, resulting in continuous depletion of vitamins and minerals that diet alone may not replenish [14,15]. Thus, multivitamin-mineral supplementation is considered necessary.

Most commonly used prenatal multivitamins contain a high iron dose (i.e. 60 mg elemental iron) and are large in tablet size. Pregnant women may experience gastrointestinal (GI) symptoms, particularly 'morning sickness', aggravated by the iron content or swallowing a large tablet [16,17]. Approximately 80% of pregnant women experience some degree of nausea and vomiting of pregnancy (NVP) which may improve towards the end of the first trimester; however, it is not uncommon for symptoms to continue beyond the first trimester or until the end of pregnancy [17,18]. Pregnant women may also experience heartburn, acid reflux, indigestion, constipation, or diarrhea, and some women may experience exacerbation of GI conditions such as irritable bowel syndrome (IBS), Crohn's disease, or ulcerative colitis. The consequence of GI irritability can deter pregnant women from starting or continuing to take prenatal multivitamins.

In a previously published study, we determined that a twice-daily, small-tablet prenatal multivitamin with a low iron dose had better tolerability than a once-daily, large-tablet prenatal multivitamin with a high iron dose [19]. It would be of interest to assess the tolerability between 2 small-size prenatal multivitamins, differing in iron content. The objective of the present study was to compare rates of adherence and reported adverse events among pregnant women who were randomized to commence supplementation with a small-tablet prenatal multivitamin, containing either a low iron dose or high iron dose.

## Methods

### Inclusion and exclusion criteria for study subjects

Women who called the Motherisk Program at The Hospital for Sick Children in Toronto were included if they were pregnant at the time of the call and had discontinued or had not started any multivitamin due to adverse events such as nausea, vomiting, constipation, diarrhea, heartburn, acid reflux, indigestion, or other GI irritability. Motherisk is a counseling program that provides information to women on the safety or risk to a developing fetus and newborn of maternal exposure to drugs, chemicals, and disease. Women were not included if their pregnancy had progressed beyond 20 weeks gestation. Women were to be excluded if they had hypersensitivities to any of the ingredients available in any of the two multivitamin formulations (either PregVit^®^, Duchesnay, Laval, Quebec; or Orifer F^®^, Sanofi-Aventis, Laval, Quebec), if they had hemochromatosis, hemosiderosis, or hemolytic anemia, or if they did not consent to the study protocol. However, no woman who was considered for the study was excluded for these reasons.

### Selection of prenatal multivitamins for study comparison

PregVit^® ^is a prenatal multivitamin that contains 35 mg elemental iron, as ferrous fumarate. The multiple vitamins and minerals are formulated into 2 small tablets (one tablet: 16 mm × 9 mm × 4 mm), thus PregVit^® ^is taken as 2 tablets per day. The 2 tablets contain different vitamins and minerals, particularly separating the iron (morning tablet) from the calcium (evening tablet) to optimize iron absorption. The use of PregVit^® ^requires a physician's prescription. Since Materna^® ^(Wyeth Pharmaceuticals, Markham, Ontario; one tablet: 18 mm × 8 mm × 8 mm; contained 60 mg elemental iron as ferrous fumarate at the time of the study) or other generic products are the most commonly used non-prescription (i.e. over-the-counter) prenatal multivitamins, they were not selected for comparison in the study because enrolled subjects who had discontinued a prenatal multivitamin most likely had discontinued any one of them. Ethically, subjects in this situation cannot be randomized to resume Materna^® ^or another generic prenatal multivitamin.

Instead, Orifer F^® ^was selected as the small-tablet (one tablet: 5 mm radius, 5 mm thickness) prenatal multivitamin, containing a high iron content (60 mg elemental iron as ferrous sulphate). It is taken daily as a single tablet and the use of Orifer F^® ^does not require a physician's prescription (i.e. over-the-counter). Comparing PregVit^® ^to Orifer F^® ^would address separation of the potential effect of iron content from that of tablet size on multivitamin tolerability among pregnant women.

### Subject recruitment and data collection

Between October 2004 and October 2006, women who called either the Motherisk General Information line or the Motherisk Nausea and Vomiting of Pregnancy (NVP) Helpline were introduced to the study by a Motherisk counselor, based on the inclusion and exclusion criteria. If the caller was interested in study participation, the counselor referred the caller to the research coordinator. The research coordinator explained the study and proceeded with enrolment after obtaining oral consent.

Based on a computer-generated randomization table, women were randomized to one of two groups. Women randomized to the '35 mg' group would commence supplementation with PregVit^® ^(low iron content, small size), and women randomized to the '60 mg' group would commence supplementation with Orifer F^® ^(high iron content, small size). An information package was mailed to each woman, instructing her to commence supplementation with her assigned prenatal multivitamin, according to the product's standard dosing (twice daily for '35 mg' group, once daily for '60 mg' group). Subjects were responsible for obtaining their own multivitamin supply through their health care providers. Information regarding the study was faxed to the physician or other health care provider of each subject.

After enrolment, subjects received a one-week follow-up telephone call and then were interviewed by telephone on a monthly basis until the end of pregnancy. Each interview documented obstetrical and medical information, adherence based on pill intake recall, and any reported adverse events. Discontinuation of the assigned multivitamin was defined as intentionally not taking the supplement and most likely not resuming the supplement. The date(s) of discontinuation at any time(s) during study participation was documented as the date(s) reported by the subject during a monthly interview.

Study completion was defined as completing monthly telephone interviews (when possible) to document pill intake and adverse events up until the end of pregnancy (i.e. 36 weeks gestation or further). Overall adherence was defined as the percentage of pill intake out of the prescribed (i.e. she ingested 200 of the 300 prescribed pills, thus her overall adherence was 67%). Standard adherence was defined as pill intake (out of the prescribed) of at least 80% (i.e. woman A ingested 82% of her pills, thus she adhered to the study intervention, while woman B ingested 67% of her pills, thus she was not adherent to study intervention). Prenatal multivitamins are intended for daily consumption during pregnancy, thus the number of pills prescribed was defined as the expected number of pills to be consumed for the assigned multivitamin for the time of study participation. For example, women in the '35 mg' group were prescribed 2 tablets per day and in a month of 30 days, were expected to take 60 tablets, while women in the '60 mg' group (although the multivitamin was non-prescription) would be expected to take 30 tablets for that same month. The study was approved by the Research Ethics Board of the Hospital for Sick Children, and all subjects gave oral, informed consent.

### Data analysis

Rates of adherence and adverse events were compared using chi-squared tests, as appropriate. Adherence was also compared between the 2 treatment groups through Kaplan-Meier survival curves in 2 ways. The first survival curve analysis compared the proportion of women with standard adherence (i.e. at least 80% pill intake over time), after having commenced supplementation with the assigned multivitamin, and the p-value was determined by the Wilcoxon (Peto-Prentice) test. The second survival curve analysis compared overall adherence among women who commenced supplementation with the assigned multivitamin, at any percentage of pill intake over time, and the p-value was determined by the log rank statistic. All curves were plotted from the coordinates of 100% (y-axis point of 1.0) at time zero (x-axis point of 0) to represent that at the beginning of the study, all subjects who commenced supplementation in each multivitamin group were adherent. Instead of fatality as the event which causes the curves to decline (as commonly used in survival curve analysis), the events were modified for the present analyses and defined as the following: a) standard adherence (i.e. at least 80% pill intake) not achieved once supplementation was initiated (first survival curve analysis), and b) discontinuation of assigned multivitamin after having commenced supplementation, at any percentage of pill intake (second survival curve analysis).

## Results

**Figure 1:**
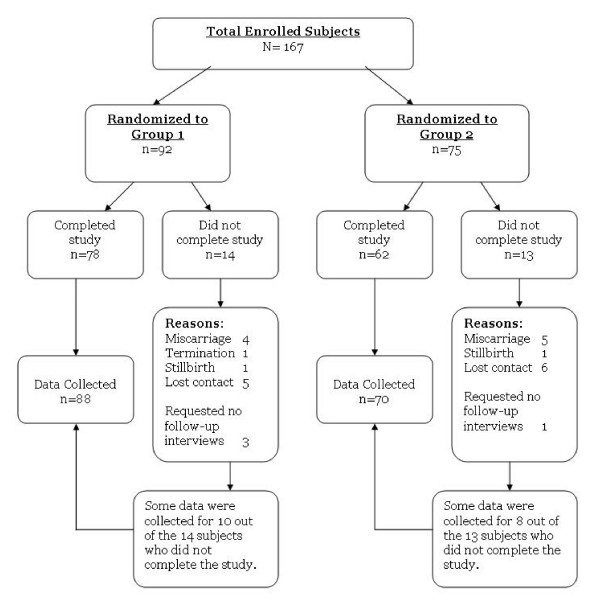
**Enrolled subjects and data collected**. Total no. of subjects who completed the study, n = 140. Total no. of subjects with (complete or partial) data collected for study, n = 158.

Between October 2004 and October 2006, 167 pregnant women were enrolled into the study through the Motherisk Program (Hospital for Sick Children), 92 women were randomized to the '35 mg' group (PregVit^®^), and 75 women were randomized to the '60 mg' group (Orifer F^®^). Although 27 women did not complete the study for various reasons (i.e. miscarriage, lost contact), some data were still collected and included in the analysis (Figure 1). Based on the inclusion criteria, 67% of subjects were enrolled because they had discontinued a previous multivitamin supplement in the current pregnancy, and the most frequently reported reasons for discontinuing or not starting to take any multivitamins were NVP (78%), tablet size or swallowing difficulties (32%), and various GI symptoms (Table 1). There was a noticeable difference between the 2 groups in terms of tablet size and constipation as the reasons reported for non-adherence to any previous multivitamins (Table 1). However, 66% of subjects in the '60 mg' group who reported issues with tablet size as a reason for prior non-adherence, still commenced supplementation with the assigned multivitamin, similarly to the 65% of subjects in the '35 mg' group who also reported prior issues with tablet size yet still commenced supplementation with the assigned multivitamin. As for the differences in previously reported constipation, it also had minimal deterrence considering that 12 of the 14 subjects (86%) in the '35 mg' group, who reported constipation as the reason for prior non-adherence, still commenced supplementation with the assigned multivitamin.

**Table 1:** Subject characteristics at time of enrolment

**Prenatal multivitamin groups, differing in iron content**			
	60 mg iron group n = 75	35 mg iron group n = 92	Total N = 167

**Study inclusion:**			

a) Had not started multivitamin in current pregnancy	27 (36%)	28 (30%)	55 (33%)
b) Had discontinued multivitamin in current pregnancy	48 (64%)	64 (70%)	112 (67%)

**Reasons for (a) or (b):**			

Nausea and/or vomiting (NVP)	62 (83%)	68 (74%)	130 (78%)
Tablet size^ψ^, swallowing difficulties, other tablet properties (i.e. taste, smell)	33 (44%)	20 (22%)	53 (32%)
Constipation^β^	4 (5%)	14 (15%)	18 (11%)
Abdominal pain, GI irritability, diarrhea, heartburn, reflux	10 (13%)	13 (14%)	23 (14%)
GI medical condition (i.e. IBS, Crohn's disease)	2 (3%)	2 (2%)	4 (2%)
Doctor's advice, not supplement taker, or lacked information	5 (7%)	6 (7%)	11 (7%)

**Maternal age (years)** Mean ± standard deviation	30 ± 5	31 ± 5	31 ± 5

**Gravidity:**			

First pregnancy	23 (31%)	20 (22%)	43 (26%)
Second or higher pregnancy	52 (69%)	72 (78%)	124 (74%)
Multivitamin intake in a previous pregnancy*	45/52 (87%)	59/72 (82%)	104/124 (84%)
Discontinued multivitamin in a previous pregnancy*	26/45 (58%)	28/59 (47%)	54/104 (52%)

*Data missing for 1 subject.

^ψ ^For the subjects who reported issues with tablet size as the reason for non-adherence with previous multivitamins, 22/33 (66%) in the '60 mg' group and 13/20 (65%) in the '35 mg' group still commenced supplementation with the assigned multivitamins.

^β ^For the subjects who reported constipation as the reason for non-adherence with previous multivitamins, 2/4 (50%) in the '60 mg' group and 12/14 (86%) in the '35 mg' group still commenced supplementation with the assigned multivitamins.

Eighty-four percent of subjects who were pregnant in the past had also supplemented with multivitamins in a previous pregnancy, and 52% of these multivitamin-takers had also discontinued the supplements in a previous pregnancy (Table 1).

**Figure 2:**
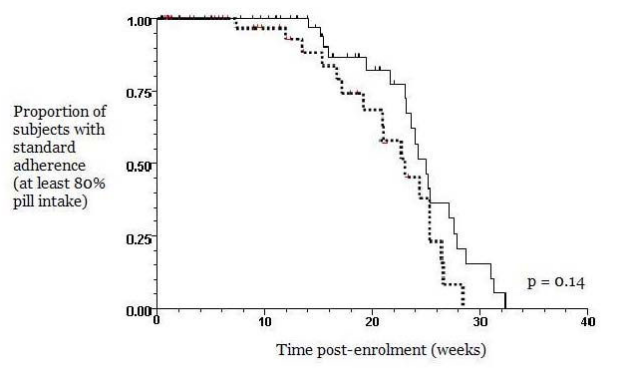
**Rates of adherence between 2 prenatal multivitamin groups, represented as Kaplan-Meier survival curves and based on the proportion of subjects with standard adherence (80% or greater pill intake), once supplementation commenced.** (Black solid line) 35 mg iron group, n = 57. (Black broken line) 60 mg iron group, n = 47.

**Figure 3:**
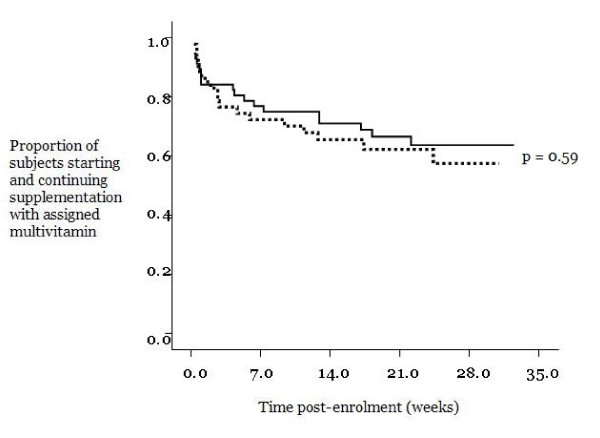
**Rates of adherence between 2 prenatal multivitamin groups, represented as Kaplan-Meier survival curves and based on the proportion of subjects who commenced and continued supplementation with assigned multivitamin (at any percentage of pill intake).** (Black solid line) 35 mg iron group, n = 57. (Black broken line) 60 mg iron group, n = 47.

A similar proportion of women in both treatment groups commenced supplementation with the assigned multivitamin (73% of '35 mg' group and 76% of '60 mg' group) (Table 2). There was no difference between the 2 groups in the gestational age at which subjects started taking the assigned multivitamins (gestational age as a mean with standard deviation, 15 ± 6 weeks for the '35 mg' group, 16 ± 7 weeks for the '60 mg' group, p = 0.57). Among those who started, only 37–38% of both groups were adherent at 80% or greater pill intake, and a slightly larger proportion of both groups had a pill intake of 50% or greater (Table 2). The range of pill intake for both groups was zero to 100%, and the mean pill intake for both groups was approximately 50%. Thus, no significant differences were detected, and any partial data collected from subjects who did not complete the study did not significantly impact results. Similarly, a large proportion of women with NVP in both groups commenced supplementation with the randomly assigned multivitamins; however, the proportions of NVP women who continued supplementation at 80% or greater pill intake was substantially lower (Table 3), with no significant difference detected between groups. Kaplan-Meier survival curves demonstrated no significant differences between the 2 groups in adherence over time, in terms of the proportion of women with standard adherence, and thus, a pill intake of at least 80% (Figure 2, p = 0.14) and in terms of the proportion of women who continued, after having commenced, supplementation with the randomly assigned multivitamin (overall adherence) (Figure 3, p = 0.59).

**Table 2:** Rates of adherence between 2 prenatal multivitamin groups, based on subjects who completed the study^β^

Prenatal multivitamin groups, differing in iron content			
	35 mg iron group (n = 78)	60 mg iron group (n = 62)	p-value (chi-squared test)

No. who started taking assigned prenatal multivitamin	57 (73%)	47 (76%)	0.86
Proportion who were ≥ 80% adherent^†^.	21/57 (37%)	18/47 (38%)	0.96
Proportion who were ≥ 50% adherent^¶^.	32/57 (56%)	28/47 (60%)	0.88

^β ^No significant difference was detected when partial data from subjects who did not complete the study were included.

^† ^Based on at least 80% pill intake.

^¶ ^Based on at least 50% pill intake

**Table 3:** Rates of adherence between 2 prenatal multivitamin groups, among subjects with nausea and vomiting of pregnancy (NVP) and study completion^β^

Prenatal multivitamin groups, differing in iron content			
	35 mg iron group (n = 78)	60 mg iron group (n = 62)	p-value (chi-squared test)

No. of subjects with NVP (at time of enrolment)	67 (86%)	56 (90%)	0.59
Proportion who started taking assigned prenatal multivitamin	49/67 (73%)	44/56 (79%)	0.63
Proportion who were ≥ 80% adherent.^ψ^	17/49 (35%)	18/44 (41%)	0.69

^β^No significant difference was detected when partial data from subjects who did not complete the study were included.

^ψ ^Based on at least 80% pill intake

There were no substantial differences in rates of reported adverse events between the 2 groups, among the pregnant women who commenced supplementation with the assigned multivitamins (Table 4).

**Table 4:** Rates of reported adverse events among pregnant women who commenced supplementation with assigned multivitamin

Adverse Events	35 mg iron group (n = 57*)	60 mg iron group (n = 47*)
No. subjects (%) who reported any adverse event(s)	27 (47%)	21 (45%)
Nausea and/or vomiting(NVP)	12 (21%)	9 (19%)
*[Proportion who reported NVP before starting assigned multivitamin]*	*[11/12]*	*[9/9]*
Constipation	13/57 (23%)	10/47 (21%)
*[Proportion who reported constipation before starting assigned multivitamin]*	*[7/13]*	*[5/10]*
Heartburn/Acid reflux	4/57 (7%)	1/47 (2%)
*[Proportion who reported heartburn/reflux before starting assigned multivitamin]*	*[2/4]*	*[1/1]*
Diarrhea	1/57 (2%)	3/47 (6%)
*[Proportion who reported diarrhea before starting assigned multivitamin]*	*[1/1]*	*[1/3]*
Abdominal pain/cramps	5/57 (9%)	5/47 (11%)
*[Proportion who reported abdominal pain or cramps before starting assigned multivitamin]*	*[1/5]*	*[2/5]*
Swallowing difficulties with tablet size	2/57 (4%)	None
*[Proportion who reported swallowing difficulties before starting assigned multivitamin]*	*[0/2]*	

*Only subjects who completed the study, with no significant difference detected when partial data from subjects who did not complete the study were included.

## Discussion

Pregnant women enrolled in the present study were those who contacted the Motherisk counseling program (Toronto, Canada), thus they were probably self-selected by heightened awareness of pregnancy health issues. Sixty-seven percent of the enrolled women had also discontinued previous multivitamin supplements in the current pregnancy due to various GI irritabilities, thus poor adherence and GI adverse events were already established among most women in the study. Although we examined a selective group of pregnant women, the findings from this present study may still be applied to a large proportion of pregnant women in the general population, considering that approximately 80% of pregnant women experience some degree of NVP or GI irritability and that prenatal multivitamin supplementation is commonly recommended in pregnancy despite these potential challenges.

The limited sample size and unequal randomization of the study is a reflection of recruiting and randomizing subjects belonging to a vulnerable population. However, the proportion of subjects who did not complete the study (i.e. drop-out) did not differ between the 2 treatment groups (15% for '35 mg' group, 17% for '60 mg' group).

Results from this randomized, prospective study of pregnant women who had not started or had discontinued previous multivitamins due to adverse events suggested that a prenatal multivitamin with a low iron dose did not improve adherence as there was no significant difference between the '35 mg' and '60 mg' groups. Although a large proportion of women in both groups commenced supplementation with the assigned multivitamins, a substantially reduced proportion of women in both groups were adherent throughout pregnancy (Tables 2). A major finding of our study was that under ideal conditions of high level of motivation and continued supervision, adherence ranged from zero to 100%, and on average women consumed only half of their pills in both groups.

Although recall of pill intake may be associated with biased reporting as it generally results in over-estimation of adherence; the interviewer encouraged subjects to be honest and emphasized that no personal judgment was placed on responses. Self-report has been commonly utilized in several studies examining intervention adherence [20-22].

Our results suggested that the low iron dose did not reduce rates of GI adverse events; however, it is important to acknowledge that the 2 multivitamin tablets differed in a number of ways – volume, shape, iron compound, and regimen – in addition to iron dose which were also considered when comparing the 2 multivitamins in terms of both adherence and GI adverse events. Both prenatal multivitamins were small tablets (relative to other common multivitamin tablet sizes), and although the shapes and volumes of the tablets differed, it did not substantially impact the issue of swallowing difficulties between the 2 groups (Table 4), and thus, no further significant differences were detected in adherence or other adverse events between the 2 groups. These results suggest that tablet size tolerability may reflect a relative perception of small tablets versus large tablets, regardless of exact volume and shape. As for the difference in iron preparations (35 mg as ferrous fumarate and 60 mg as ferrous sulphate), it also had a negligible impact as no significant differences were detected in adherence or GI adverse events between the 2 groups, which is consistent with several studies that examined GI intolerance with respect to iron content (i.e. doses, compounds) [16,23-25].

It is of interest that with the '35 mg' group, the prenatal multivitamin is taken as 2 tablets per day, separating the iron from calcium and hence preventing their interaction, and allowing a lower dose of elemental iron (35 mg) to yield similar systemic exposure as 60 mg taken with calcium [26]. Despite having to take 2 tablets per day, overall adherence with the '35 mg' group was not lower compared to the once-per-day '60 mg' group. This finding is consistent with the results of a systematic review showing no differences in adherence between once versus twice daily administration, with a significant decrease in adherence only with three doses per day [27].

Multivitamin tolerability depends not only on the supplement itself, but also on whether or not pregnant women suffer from and properly manage NVP symptoms. Similar to the general population of pregnant women, most of the pregnant women in both treatment groups experienced NVP and this may well explain why overall adherence under optimal conditions was not predominantly higher than 50% pill intake.

## Conclusion

These new findings must be considered when planning public health strategies to ensure folate and other vitamin and mineral supplementation throughout pregnancy. If prenatal multivitamin formulation remains in the present state, adherence among pregnant women, even with optimal motivation and guidance, may not significantly improve above 50% pill intake; however, this present study demonstrated that potential improvements may be associated with tablet size.

## Competing interests

The authors declare that they have no competing interests.

## Authors' contributions

PN drafted the forms and questionnaires for data collection, enrolled study subjects, conducted telephone interviews, collected and analyzed data, and drafted the manuscript. AN–O contributed to data analysis, particularly the survival curve analysis, and manuscript editing. AL contributed to data analysis, particularly the survival curve analysis, and manuscript editing. DLO'C contributed to drafting and editing the manuscript. TRE contributed to data analysis and manuscript editing. AT contributed to data analysis. GK conceived of the study and participated in its design and coordination, and contributed to manuscript editing. All authors read and approved the final manuscript. The study was supported by a grant from Duchesnay Inc., Laval, Quebec, Canada.

## Pre-publication history

The pre-publication history for this paper can be accessed here:


